# Tim-3 signaling blockade with α-lactose induces compensatory TIGIT expression in *Plasmodium berghei* ANKA-infected mice

**DOI:** 10.1186/s13071-019-3788-x

**Published:** 2019-11-11

**Authors:** Yiwei Zhang, Ning Jiang, Ting Zhang, Ran Chen, Ying Feng, Xiaoyu Sang, Na Yang, Qijun Chen

**Affiliations:** 1 Key Laboratory of Livestock Infectious Diseases in Northeast China, Ministry of Education, College of Animal Science and Veterinary Medicine, Shenyang Agricultural University, Shenyang, 110866 China; 2 The Research Unit for Pathogenic Mechanisms of Zoonotic Parasites, Chinese Academy of Medical Sciences, 120 Dongling Road, Shenyang, 110866 China

**Keywords:** *Plasmodium berghei*, Tim-3, Immune escape, Cytokine, α-lactose, TIGIT

## Abstract

**Background:**

Malaria, one of the largest health burdens worldwide, is caused by *Plasmodium* spp. infection. Upon infection, the host’s immune system begins to clear the parasites. However, *Plasmodium* species have evolved to escape the host’s immune clearance. T-cell immunoglobulin and mucin domain 3 (Tim-3), a surface molecule on most immune cells, is often referred to as an exhaustion marker. Galectin (Gal)-9 is a Tim-3 ligand and the T helper (Th) 1 cell response is inhibited when Gal-9 binds to Tim-3. In the present study, dynamic expression of Tim-3 on key populations of lymphocytes during infection periods of *Plasmodium berghei* and its significance in disease resistance and pathogenesis were explored.

**Methods:**

Tim-3 expression on critical lymphocyte populations and the proportion of these cells, as well as the levels of cytokines in the sera of infected mice, were detected by flow cytometry. Further, *in vitro* anti-Tim-3 assay using an anti-Tim-3 antibody and *in vivo* Tim-3-Gal-9 signaling blockade assays using α-lactose (an antagonist of Gal-9) were conducted. An Annexin V Apoptosis Detection Kit with propidium iodide was used to detect apoptosis. In addition, proteins associated with apoptosis in lung and spleen tissues were confirmed by Western blotting assays.

**Results:**

Increased Tim-3 expression on splenic CD8^+^ and splenic CD4^+^, and circulatory CD4^+^ T cells was associated with a reduction in the proportion of these cells. Furthermore, the levels of interleukin (IL)-2, IL-4, IL-6, IL-22, and interferon (IFN)-γ, but not that of tumor necrosis factor alpha (TNF-α), IL-10, and IL-9, increased to their highest levels at day 4 post-infection and decreased thereafter. Blocking Tim-3 signaling *in vitro* inhibited lymphocyte apoptosis. Tim-3-Gal-9 signaling blockade *in vivo* did not protect the mice, but induced the expression of the immunosuppressive molecule, T cell immunoreceptor with Ig and ITIM domains (TIGIT), in *Plasmodium berghei* ANKA*-*infected mice.

**Conclusions:**

Tim-3 on lymphocytes negatively regulates cell-mediated immunity against *Plasmodium* infection, and blocking Tim-3-galectin 9 signaling using α-lactose did not significantly protect the mice; however, it induced the compensatory expression of TIGIT. Further investigations are required to identify whether combined blockade of Tim-3 and TIGIT signaling could achieve a better protective effect.
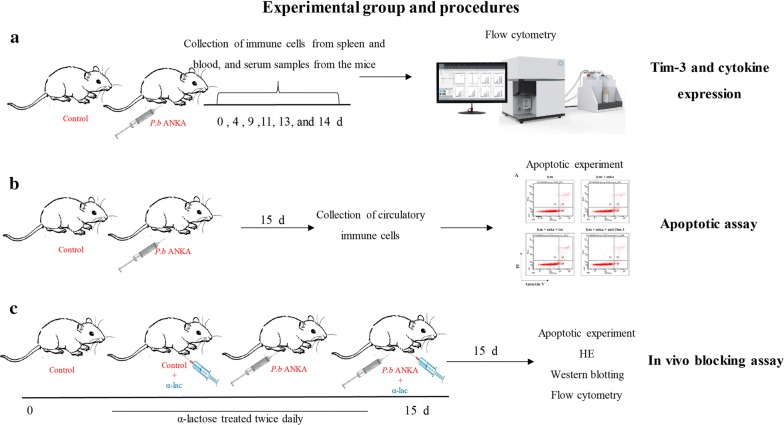

## Background

Malaria, which is caused by infection with *Plasmodium* species, is one of the biggest global health burdens worldwide and caused about 219 million clinical cases and 435,000 deaths in 2017 [1]. *Plasmodium* spp. trigger an array of signals and responses in both the innate and adaptive immune systems of the host to control the parasite, which can prevent the onset of severe disease under certain circumstances [2]. Meanwhile, to survive in the host, parasites execute a series of immune escape strategies, including antigenic variation, polymorphism, and the expression of inhibitory molecules on the surface of immune cells [3].

In the erythrocytic stage of infection, antibodies from malaria-exposed individuals can provide protective immunity by promoting opsonic phagocytosis of merozoites and inducing monocyte activation and pro-inflammatory cytokine production [4]. Antibodies can also bind to extracellular merozoites to prevent the invasion of erythrocytes and enhance complement fixation on merozoites, which mark merozoites for lysis by complement activation and have significantly greater invasion-inhibitory activity in the presence of complement [5]. Previous studies have found substantial levels of major histocompatibility (MHC) class I molecules expressed on erythroblasts before or after *Plasmodium* infection, and CD8^+^ T cells might target MHC I-positive parasitized erythroblasts and produce interferon gamma (IFN-γ) to clear the parasites [6]. CD4^+^ T cells, which are essential for balancing protection and pathology of hosts, are major producers of pro-inflammatory and regulatory cytokines [7]. T follicular helper (TFH) cells (a subset of CD4^+^ T cells) release interleukin (IL)-21, which is a key to promoting effective germinal center formation and to activate protective, long-lasting B cell responses and humoral immune responses [8]. Other components, such as natural killer (NK) cells, γδT cells, and the host microbiota, are also involved in the clearance of parasites, directly or indirectly [9].

Unfortunately, *Plasmodium* can avoid clearing by host cells through a series of immune escape strategies. For example, the formation of rosettes, the binding of uninfected erythrocytes around an infected erythrocyte, can help *Plasmodium* to escape clearance [10]. Furthermore, expression of variable protein families on the infected erythrocyte surface, such as *Plasmodium falciparum* erythrocyte membrane protein 1 (PfEMP1), will help the parasites to evade immune recognition [11]. Notably, *P. falciparum* infection always hijacks the host’s immune system by activating checkpoint inhibitor molecules, resulting in immune exhaustion [12]. A study showed that the expression levels of exhaustion markers, such as T-cell immunoglobulin and mucin domain 3 (Tim-3), lymphocyte activation gene-3 (LAG-3), cytotoxic T-lymphocyte-associated protein-4 (CTLA-4), and programmed death-1 (PD-1), were higher in γδ and total T cells from *P. vivax*-infected patients than in those from uninfected individuals [13]. PD-1 is a well-known inhibitory molecule expressed by T cells and B cells [14]. During *Plasmodium* infection, PD-1 is upregulated in parasite-specific CD4^+^ and CD8^+^ T cells [15]. PD-1 expression on parasite-specific CD4^+^ cells results in a reduction in proliferation, as well as IFN-γ and tumor necrosis factor alpha (TNF-α) secretion, during the chronic phase of malaria [16]. Blocking CTLA-4 enhanced the production of IFN-γ from liver-derived T cells upon *Plasmodium* infection [17]. LAG-3 was reported to be expressed on dysfunctional or exhausted parasite-specific CD4^+^ T cells during malaria infection, and the combined blockade of LAG-3 and PD-L1 inhibitory molecules using antibodies improved CD4^+^ T-cell functions and accelerated parasite clearance [18]. A previous study showed the important regulatory function of Tim-3, in which, patients with cancer, resistance to anti-PD-1 therapy was prevented when an anti-Tim-3 antibody was administered together with an anti-PD-1 agent [19]. Therefore, in-depth analysis on the functions of Tim-3 is necessary. We have previously found that the expression of Tim-3 was induced and Tim-3 signaling blockade using an anti-Tim-3 antibody restored lymphocyte activity and accelerated parasite clearance [20]. Galectin (Gal) 9 was the first reported ligand for Tim-3 and engagement of Tim-3 by Gal-9 leads to Th1 cell death [21]. A previous study showed that α-lactose, which is more convenient to obtain and cheaper than anti-Tim-3 antibody, is an antagonist of Gal-9 that could successfully block Gal-9 binding to Tim-3 [22].

In the present study, we aimed to analyze the expression of Tim-3 on critical splenic and circulatory lymphocyte populations and the proportion of these cells, as well as certain cytokines in the sera of mice during malaria infection. In addition, the regulatory role of Tim-3 in *Plasmodium* infection and the effect of Tim-3-Gal-9 signaling blockade by α-lactose in disease resistance and pathogenesis of *P. berghei* ANKA-infected mice were explored.

## Methods

### Animals

Female BALB/c mice of N phenotype (6–8 weeks-old) were purchased from Liaoning Changsheng Biological Technology Company, Benxi, China and maintained in a pathogen-free facility. Free access to water and food was provided to animals during the experiments.

### *Plasmodium berghei* infection and study design

To detect the changes in Tim-3 expression on critical splenic and circulatory lymphocyte populations during infection, 42 mice were randomly divided into six groups (*n *= 7 per group). Each mouse was infected intraperitoneally (i.p.) by injection with 10^5^ infected red blood cells (iRBCs) of *P. berghei* ANKA (PbA). The mice were sacrificed at 0, 4, 9, 11, 13 and 14 days post-infection (p.i.) (Additional file 1: Figure S1a). Mice spleen tissues were obtained to calculate spleen indices (Spleen index= spleen weight (mg) × 10/body weight (g) × 100%).

For the apoptotic assay, mice were divided into two groups (*n *= 7 per group): the control group (healthy mice) and the PbA group (mice injected i.p. with 10^5^ iRBCs of PbA). All mice were sacrificed at 15 days p.i. (Additional file 1: Figure S1b).

In the α-lactose (α-lac) (Sigma-Aldrich, St. Louis, MO, USA) treatment experiment, mice were divided into several groups, some of which were injected i.p. with 100 µl of 450 mM or 550 mM of sterile-filtered α-lac solution, twice daily, starting from day 1 p.i. until the day the mice were sacrificed (Additional file 1: Figure S1c).

### Cell preparation and flow cytometry analysis

Splenic single cell suspension and circulatory immune cells were obtained as previously described [23]. The antibodies used in this study were as follows: Pacific Blue anti-mouse CD45; allophycocyanin-conjugated (APC) anti-mouse CD3; fluorescein isothiocyanate-conjugated (FITC) anti-mouse CD8a; peridinin chlorophyll protein complex-conjugated (PerCP) anti-mouse CD4; Brilliant Violet 510™-conjugated (BV510) anti-mouse CD19; phycoerythrin/Cyanine7-conjugated (PE/Cy7) anti-mouse CD49b; PE/Cy7 anti-mouse CD226; PE anti-mouse CD366 (Tim-3); PE anti-mouse T cell immunoreceptor with Ig and ITIM domains (TIGIT); Pacific Blue Rat immunoglobulin (Ig)G2b, κ Isotype control (Ctrl); APC Rat IgG2b, κ Isotype Ctrl; FITC Rat IgG2a, κ Isotype Ctrl; PerCP Rat IgG2b, κ Isotype Ctrl; PE Rat IgG2a, κ Isotype Ctrl; BV510 Rat IgG2a, κ Isotype Ctrl; and PE/cy7 Armenian Hamster IgG Isotype Ctrl (all from Biolegend, San Diego, CA, USA). The cells were detected and analyzed using a fluorescence activated cells sorting (FACS) Aria III flow cytometer (BD Biosciences, San Jose, CA, USA), and the gates for positive cells were defined using the isotype and fluorescence minus one (FMO) controls.

### Cytokine detection

Levels of IL-2, IL-4, IL-6, IL-9, IL-10, IL-22, TNF-α, and IFN-γ in mouse serum were determined using a LEGENDplex Mouse Th Cytokine Panel (BioLegend). Samples were assayed according to the BioLegend standard protocol and were examined using FACSAria III (BD Biosciences) driven by the FACSDiva software (BD Biosciences). The detection limitations of the cytokines were as follows: IL-6, IL-9, IL-10, and IFN-γ were > 0 pg/ml; TNF-α was > 3.26 pg/ml; IL-2 was > 2.99 pg/ml; IL-4 was > 1.52 pg/ml; and IL-22 was > 2.24 pg/ml.

### Apoptotic assay

Single murine circulatory cells were isolated using Histopaque-1083 (Sigma-Aldrich) according to the manufacturer’s instructions. Then 10^6^ single cells were stained with Pacific Blue anti-mouse CD45, FITC anti-mouse CD8a, and BV510 anti-mouse CD19. After staining, the cells were stained with an APC Annexin V Apoptosis Detection Kit with propidium iodide (PI; Biolegend) according to the manufacturer’s instructions. Stained cells were analyzed immediately by flow cytometry.

### *In vitro* anti-Tim-3 treatment assay

Murine circulatory lymphocytes isolated from healthy mice were cultured in Roswell Park Memorial Institute (RPMI) 1640 medium (HyClone, Waltham, MA, USA) supplemented with 10% fetal bovine serum (Gibco, Carlsbad, CA, USA) and plated at 5 × 10^5^ cells per well in 96-well polystyrene plates. Then, 5 μg/ml of the anti-mouse Tim-3 antibody (Biolegend) were added and incubated for 30 min to block the Tim-3 signaling pathway. Purified rat IgG2α κ isotype (Biolegend) was used as the control antibody. PbA-infected erythrocytes with a parasitemia > 30% were then added into the indicated wells at a concentration of 1 × 10^5^ per well. The cells were incubated for 24 h and collected afterwards for the apoptotic assay using a FITC Annexin V Apoptosis Detection Kit with PI (Biolegend).

### *In vivo* signaling blockade

To block Gal-9 binding to Tim-3, α-lactose was used [22, 24]. Mice were divided into six groups: (i) Control group (healthy mice); (ii) Control-450 mM α-lactose group (healthy mice treated with 450 mM of α- lactose); (iii) Control-550 mM α-lactose group (healthy mice treated with 550 mM of α- lactose); (iv) PbA group (mice infected with 10^5^ iRBCs i.p.); (v) PbA-450 mM α- lactose group (mice infected with 10^5^ iRBCs i.p., and treated with 450 mM of α- lactose); and (vi) PbA-550 mM α- lactose group (mice infected with 10^5^ iRBCs i.p., and treated with 550 mM of α- lactose). There were 10 mice in each group.

According to the survival rates, the group treated with 550 mM of α- lactose was chosen for further examination. At 15 days p.i. and 12 h after the last treatment, the mice were sacrificed, and the isolated lung and spleen tissues were obtained for hematoxylin-eosin staining (HE) and Western blotting assays. Circulatory immune cells were isolated using Histopaque-1083 (Sigma-Aldrich) for flow cytometry and apoptotic assays.

### Histopathological analysis of spleen and lung tissue

Mice were euthanized at 15 days p.i. Spleen and lung tissues were embedded in 4% paraformaldehyde (PFA), and serial 5-μm cryosections were obtained and stained with HE. After dehydration, the histological samples were examined under a Leica microscope (Leica Microsystem, Wetzlar, Germany). Lung [25] and spleen [26] injury scoring systems were used to measure the extent of tissue damage. The area of hemosiderin was measured by Image J software (National Institutes of Health, Bethesda, MD, USA).

### Western blotting assay

Total proteins of mouse lung and spleen tissues were obtained using lysis buffer (Shenggong, Shanghai, China) according to the manufacturer’s instructions. Western blotting was performed as described previously [27]. Briefly, total proteins from each sample were separated using sodium dodecylsulfate polyacrylamide gel electrophoresis (SDS-PAGE) and transferred onto polyvinylidene difluoride membranes (Bio-Rad, Redmond, WA, USA). After blocking with 5% non-fat milk (wt/vol) in phosphate-buffered saline (PBS) with Tween-20 (PBST) for 1 h at 37 °C, each membrane was incubated in PBST with anti-cleaved-caspase 3 monoclonal antibodies (CST, Trask Lane Danvers, MA, USA), anti-BAX (BCL2 associated X, apoptosis regulator) monoclonal antibodies (Biolegend), or anti-β-actin antibodies (CST) overnight at 4 °C. This was followed by washing and subsequent incubation with horseradish peroxidase (HRP)-conjugated Goat Anti-Rabbit IgG secondary antibodies (Beyotime Biotechnology, Shanghai, China). All analyses were performed in duplicate. The membranes were scanned using the Azure c300 system (Azure Biosystems, Dublin, CA, USA) and the signals were quantified using ImageJ software. Target protein levels were normalized to that of β-actin.

### Data analysis

All analyses were carried out using GraphPad Prism 6 (GraphPad Software, Inc., La Jolla, CA, USA). The results were analyzed using a two-tailed Student’s t-test or an ANOVA test when there were comparisons between multiple groups. Results were expressed as the mean ± SD. A *P-*value of less than 0.05 was considered statistically significant. Cytokine calculations were performed using the LEGENDplex 8.0 application (VigeneTech Inc., Carlisle, MA, USA).

## Results

### *Plasmodium berghei* ANKA infection induced an increased Tim-3 expression and a reduction in the splenic lymphocyte population

**Fig. 1:**
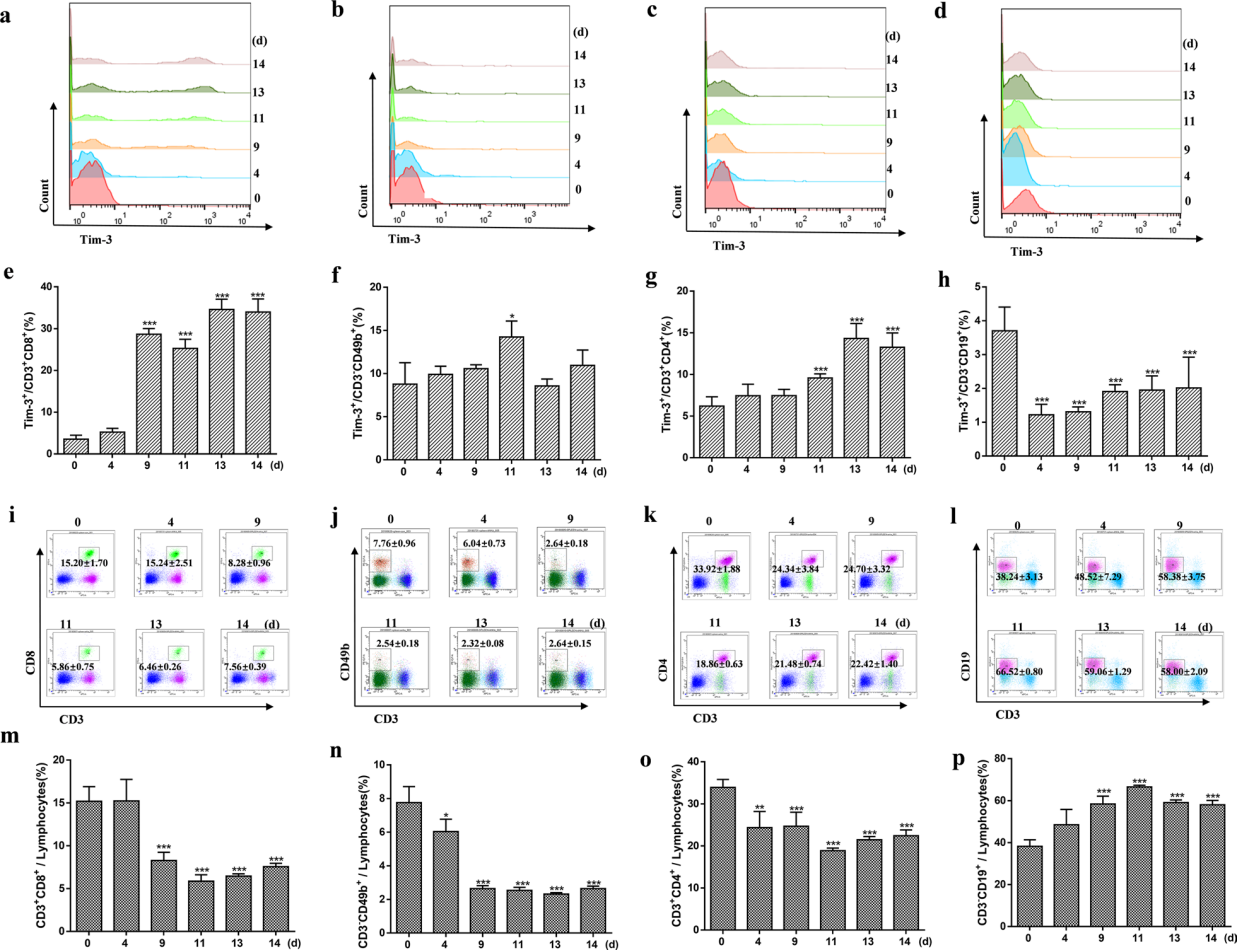
Expression of Tim-3 on splenic CD3^+^CD8^+^, CD3^-^CD49b^+^, CD3^+^CD4^+^ and CD3^−^CD19^+^ cells, and the proportional changes in *P. berghei*-infected mice. Tim-3 expression on splenic CD3^+^CD8^+^ T cells (**a**, **e**), CD3^-^CD49b^+^ cells (**b**, **f**), CD3^+^CD4^+^ cells (**c**, **g**), CD3^−^CD19^+^ cells (**d**, **h**) and the proportions of these cells (i-p) were detected by flow cytometry at days 0, 4, 9, 11, 13, and 14 p.i. (**a**–**d**) Representative FACS plots of Tim-3 expression on CD3^+^CD8^+^ T cells, CD3^−^CD49b^+^ cells, CD3^+^CD4^+^ T cells and CD3^−^CD19^+^ cells. (**e**–**h**) Histograms showing the proportion of Tim-3^+^ cells within the CD3^+^CD8^+^ T cells, CD3^−^CD49b^+^ cells, CD3^+^CD4^+^ T cells and CD3^−^CD19^+^ cells. (**i**–**l**) Representative FACS plots of CD3^−^CD49b^+^ cells, CD3^+^CD8^+^ T cells, CD3^+^CD4^+^ T cells and CD3^−^CD19^+^ cells in splenic lymphocytes. (**m**–**p**) Histograms showing the proportion of CD3^−^CD49b^+^ cells, CD3^+^CD8^+^ T cells, CD3^+^CD4^+^ T cells and CD3^−^CD19^+^ cells in splenic lymphocytes. The results are representative of three independent experiments with five to seven mice in each group per experiment, with data denoting means ± SDs. *P < 0.05, **P < 0.01, ***P < 0.001, *indicates comparisons with the day 0 group

**Fig. 2:**
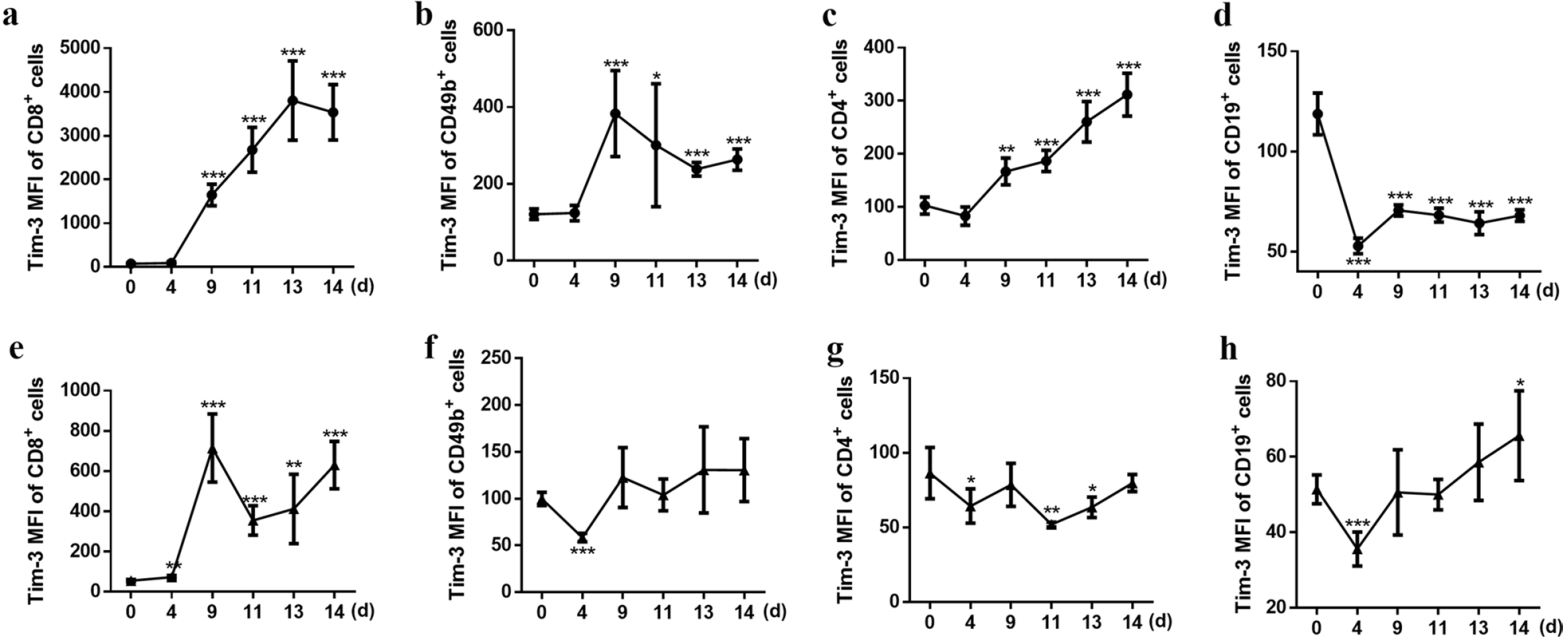
Tim-3 MFI of critical splenic and circulatory lymphocytes in mice infected with *P. berghei* ANKA. (**a**–**d**) Tim-3 MFI of splenic immune cells. (**e**–**h**) Tim-3 MFI of circulatory immune cells. The results are representative of three independent experiments with five to seven mice in each group per experiment, with data denoting means ± SDs. *P < 0.05, **P < 0.01, ***P < 0.001, *indicates comparisons with the day 0 group

Tim-3 has been previously reported to play an important role in *Plasmodium* infection in human samples and C57BL/6 mice [20]. Here, the expression of Tim-3 on splenic lymphocyte populations was investigated in BALB/c mice infected with PbA. Tim-3 expression on splenic CD8^+^ T cells (Fig. 1a, e, ANOVA: *F*_(5, 24)_= 243.822, *P *< 0.0001), CD49b^+^ cells (Fig. 1b, f, ANOVA: *F*_(5, 24)_= 8.833, *P *< 0.0001), and CD4^+^ T cells (Fig. 1c, g, ANOVA: *F*_(5, 24)_= 33.384, *P *< 0.0001) gradually increased during the infection periods. However, a reduction in the expression of Tim-3 on splenic CD19^+^ cells (Fig. 1d, h, ANOVA: *F*_(5, 35)_= 18.549, *P *< 0.0001) was observed. This followed with decreased proportions of CD8^+^ T cells (Fig. 1i, m, ANOVA: *F*_(5, 24)_= 50.992, *P *< 0.0001), CD49b^+^ cells (Fig. 1j, n, ANOVA: *F*_(5, 24)_= 104.871, *P *< 0.0001), and CD4^+^ T cells (Fig. 1k, o, ANOVA: *F*_(5, 24)_= 24.893, *P *< 0.0001) p.i., while the proportion of splenic B cells significantly increased from day 4 after infection (Fig. 1l, p, ANOVA: *F*_(5, 24)_= 35.299, *P *< 0.0001). Furthermore, the Tim-3 median fluorescence intensity (MFI) of CD8^+^ T cells (Fig 2a), CD49b^+^ cells (Fig 2b), and CD4^+^ T cells (Fig 2c) also increased compared with that of the day 0 group. But the Tim-3 MFI of CD19^+^ cells was reduced (Fig. 2d).

### Increased expression of Tim-3 on circulatory CD4^+^ T cells was associated with the reduction of cell numbers

**Fig. 3:**
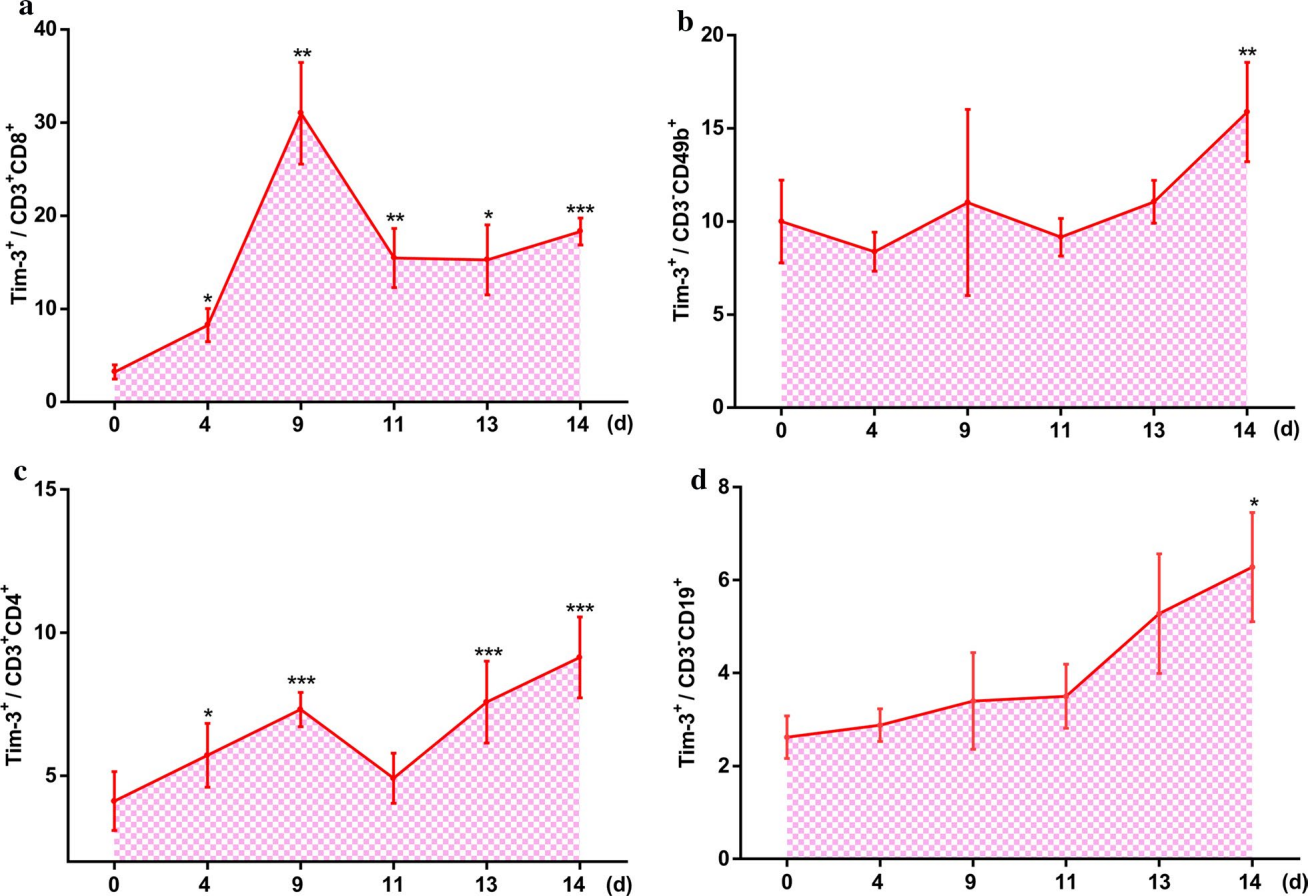
The expression of Tim-3 on circulatory lymphocytes. Tim-3 expression on circulatory CD3^+^CD8^+^ T cells (**a**), CD3^−^CD49b^+^ cells (**b**), CD3^+^CD4^+^ cells (**c**), CD3^−^CD19^+^ cells (**d**) were detected by flow cytometry at days 0, 4, 9, 11, 13, and 14 p.i. The results are representative of three independent experiments with five to seven mice in each group per experiment, with data denoting means ± SDs. *P < 0.05, **P < 0.01, ***P < 0.001, *indicates comparisons with the day 0 group

The expression of Tim-3 on circulatory lymphocyte populations was detected after mice were infected with PbA (Fig. 3a–d). Tim-3 expression on circulatory CD8^+^ and CD4^+^ T cells increased (Fig. 3a, c), while only the Tim-3 MFI of circulatory CD8^+^ T cells increased (Fig. 2e). Meanwhile, there was no significant difference in the expression of Tim-3 on circulatory CD49b^+^ and CD19^+^ cells (Fig. 3b, d), except on day 14, at which point Tim-3 expression was increased as compared with that in the day 0 group.

The proportions of different lymphocyte populations obtained from the peripheral blood of mice were also analyzed by flow cytometry. An increasing trend of circulatory CD8^+^ T cells (Additional file 1: Figure S2a, c) and a decreasing trend of circulatory CD49b^+^ cells (Additional file 1: Figure S2b, d) was observed. However, the increasing trend in the proportion of circulatory CD8^+^ T cells was not significant (Additional file 1: Figure S2c). In addition, the proportion of circulatory CD4^+^ T cells showed a decreasing trend (Additional file 1: Figure S3a, c) as Tim-3 expression increased on these cells (Fig. 3c).

### Splenomegaly was observed post-infection

Post-infection, splenomegaly occurred, and spleen indices increased (Additional file 1: Figure S4). Thus, the proportions of different populations of splenic and circulatory immune cells were analyzed (Additional file 1: Figure S5). The proportions of CD4^+^, CD8^+^, CD19^+^ and CD49b^+^ cells decreased and other immune cell types (including monocytes, dendritic cells (DCs), and macrophages, but not lymphocytes) increased in both the spleen (Additional file 1: Figure S5a) and the circulation (Additional file 1: Figure S5b).

### Pro-inflammatory cytokines in mouse sera increased to the highest levels at the early stage of infection

Cytokines play an important role in *Plasmodium* infection [28] and the levels of cytokines are associated with the expression of Tim-3 [29]. Thus, the presence of IL-2, IL-4, IL-6, IL-9, IL-10, IL-22, TNF-α, and IFN-γ in the mouse sera at days 4, 9, 11, 13 and 14 p.i. was determined quantitatively (Additional file 1: Figure S6a–h). The levels of IL-4 (Additional file 1: Figure S6a), IL-6 (Additional file 1: Figure S6b), IL-22 (Additional file 1: Figure S6c), IFN-γ (Additional file 1: Figure S6d), and IL-2 (Additional file 1: Figure S6f) increased at 4 days p.i., and decreased afterwards. The level of TNF-α (Additional file 1: Figure S6e) and IL-10 (Additional file 1: Figure S6g) gradually increased. No significant changes in IL-9 level was observed (Additional file 1: Figure S6h).

### The proportion of apoptotic lymphocytes increased post-infection and anti-Tim-3 treatment protected lymphocytes from apoptosis during *P. berghei* ANKA infection *in vitro*

**Fig. 4:**
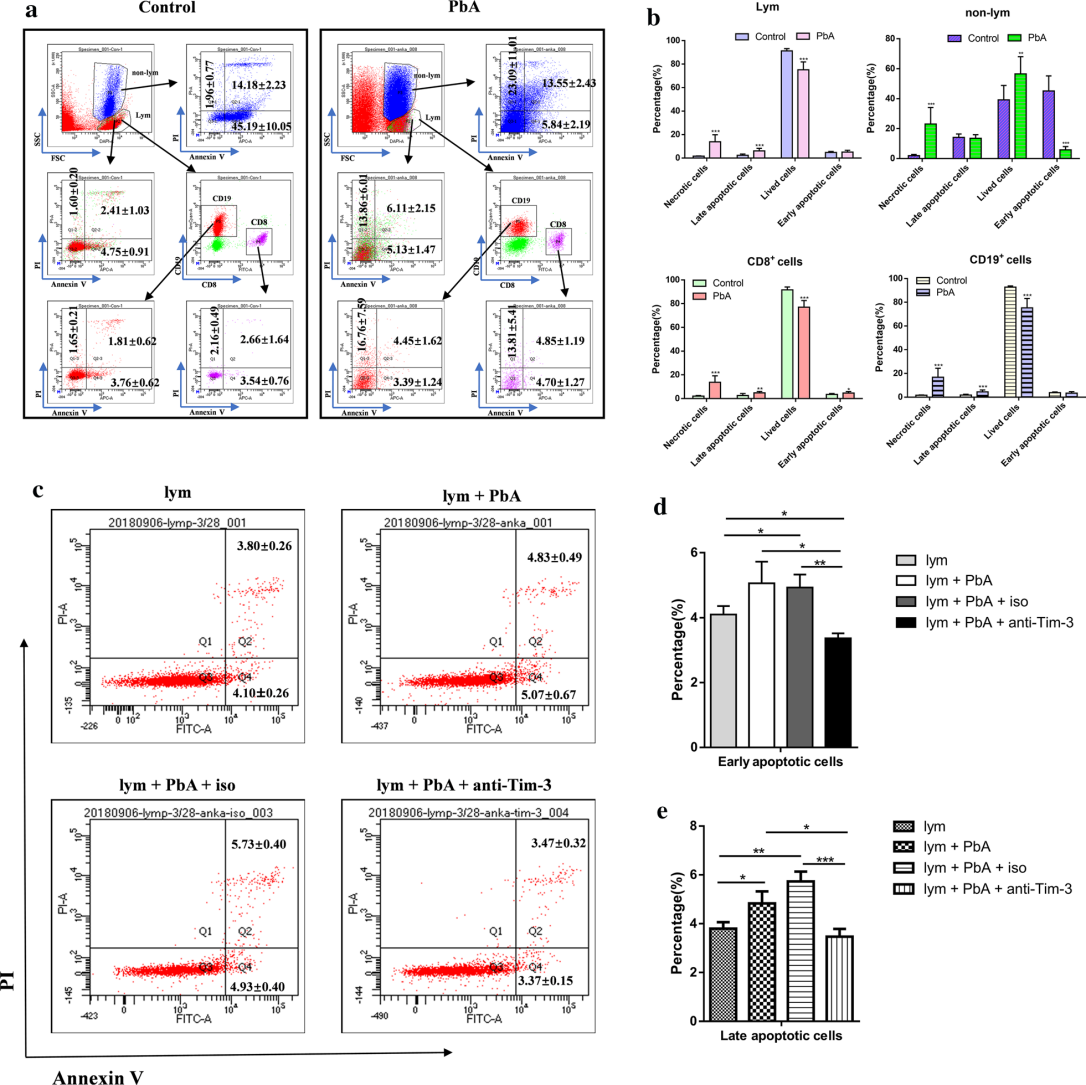
*Plasmodium berghei* ANKA infection induced apoptosis in mice lymphocytes and anti-Tim-3 treatment protected lymphocytes from apoptosis *in vitro.* (**a**, **b**) Necrotic and apoptotic circulatory immune cells at 15 days p.i. The gate strategy of propidium iodide (PI)-positive and annexin V-negative (Q1), PI- and annexin V-positive (Q2), PI- and annexin V-negative (Q3), and PI-negative and annexin V-positive (Q4) cells in lymphocytes (the dot plots marked in red and named lym), non-lymphocytes (the dot plots marked in blue and named non-lym), CD8^+^ T cells (the dot plots marked in purple and named Tc) and CD19^+^ cells (the dot plots marked in red and named B) populations. Histograms comparing the proportion of different kinds of apoptotic cells in lymphocytes, non-lymphocytes, CD8^+^ cells, and CD19^+^ cells. (**c**, **d**, and **e**) Circulatory lymphocytes from healthy mice were incubated with 1 × 10^5^ PbA-infected erythrocytes in the presence of 5 µg/ml of an anti-Tim-3 antibody (lym + PbA + anti-Tim-3), or rat IgG2α κ isotype (lym + PbA + iso), and circulatory lymphocytes from healthy mice were incubated with 1 × 10^5^ PbA-infected erythrocytes as a negative control (lym + PbA), with circulatory lymphocytes from healthy mice as the blank control (lym). The cells were collected after cultivation for 24 h and analyzed by flow cytometry. The gating strategy of PI-positive and annexin V-negative (Q1), PI- and annexin V-positive (Q2), PI and annexin V-negative (Q3), and PI-negative and annexin V-positive (Q4) cells in lymphocytes. Histograms comparing the proportion of early apoptotic cells (Q4). Histograms comparing the proportion of late apoptotic cells (Q2). The results are representative of three independent experiments with five to seven mice in each group per experiment, with data denoting means ± SDs. *P < 0.05, **P < 0.01, ***P < 0.001. *Abbreviations*: PbA, *P. berghei* ANKA; Lym, lymphocytes

The apoptosis of immune cell populations in PbA-infected mice was investigated. Single murine circulatory cells from PbA-infected mice were isolated and stained with anti-mouse CD45, anti-mouse CD8a, anti-mouse CD19, Annexin V-APC, and PI. Single circulatory cells from healthy mice were treated as controls. At 15 days p.i., the ratio of necrotic cells significantly increased in lymphocytes (Fig. 4a, b, *t*_(14)_= 5.770, *P *< 0.0001), non-lymphocytes (Fig. 4a, b, *t*_(14)_= 5.415, *P *< 0.0001), CD8^+^ T (Fig. 4a, b, *t*_(14)_= 6.065, *P *< 0.0001) cells, and CD19^+^ cells (Fig. 4a, b, *t*_(14)_= 5.632, *P *< 0.0001). The ratio of late apoptotic cells increased in lymphocytes (Fig. 4a, b, *t*_(14)_= 4.398, *P *= 0.0006), CD8^+^ T cells (Fig. 4a, b, *t*_(14)_= 3.056, *P *= 0.008), and CD19^+^ cells (Fig. 4a, b, *t*_(14)_= 4.314, *P *= 0.0007). The ratio of early apoptotic cells increased only in CD8^+^ T cells (Fig. 4a, b, *t*_(14)_= 2.223, *P *= 0.04); however, the ratio of early apoptotic cells decreased in the non-lymphocytes (Fig. 4a, b, *t*_(14)_= -10.820, *P *< 0.0001).

To further investigate whether the increased expression of Tim-3 plays a role in the loss of lymphocytes during *P. berghei* infection in BALB/c mice, murine circulatory lymphocytes were treated with an anti-Tim-3 antibody or a rat IgG2α K isotype antibody during 24 h of co-culture with PbA *in vitro*. The ratios of early and late apoptotic lymphocytes were significantly lower in the group treated with anti-Tim-3 antibodies than in other groups (Fig. 4c-e, ANOVA: *F*_(3, 8)_= 10.756, *P *= 0.004 in the early apoptotic lymphocytes; ANOVA: *F*_(3, 8)_= 21.952, *P *< 0.0001 in the late apoptotic lymphocytes). These results indicated that the upregulation of Tim-3 might participate in the loss of circulatory lymphocytes during *Plasmodium* infection.

### α-Lactose did not alleviate hemosiderosis in lung and spleen tissues in *P. berghei* ANKA-infected mice

**Fig. 5:**
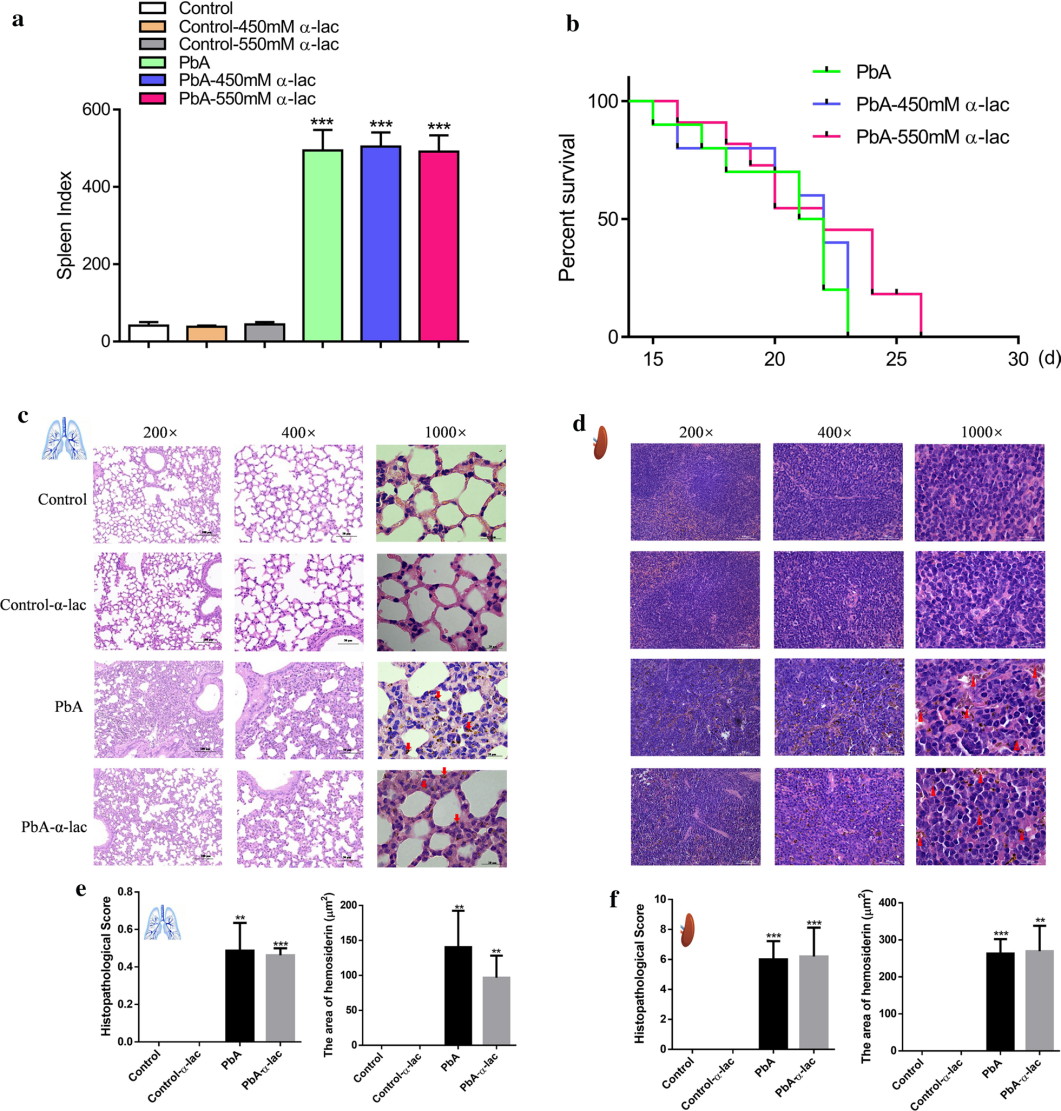
Spleen index, survival rate, and the histopathology of lungs and spleens in *P. berghei*-infected mice with or without α-lactose treatment. **a** At 15 days p.i., mice were sacrificed and spleen indices were calculated. Spleen index= spleen weight (mg) × 10/body weight (g) × 100%. **b** Survival rate. *P. berghei*-infected mice (*n  *= 10) and those treated with 450 mM α-lactose (*n  *= 10) died between days 15 and 23 p.i. *P. berghei*-infected mice treated with 550 mM α-lactose (*n * = 10) died between days 16 and 26 p.i. **c** Histopathological changes in the lung tissues detected using HE staining. **d** Histopathological changes in the spleen tissues detected using HE staining. **e** Histograms of the histopathological score and the area of hemosiderin in lung tissue. **f** Histograms of the histopathological score and the area of hemosiderin in spleen tissue. The results are representative of three independent experiments with five to seven mice in each group per experiment, with data denoting means ± SDs. *P < 0.05, **P < 0.01, ***P < 0.001. *indicates comparisons with the control group

α-Lactose is usually used to block Gal-9-Tim-3 signaling [22]. The spleen indices of the mice increased 15 days p.i. in the PbA-, PbA-450 mM α-lactose-, and PbA-550 mM α-lactose-treated group; however, there were no significant differences among the three groups (Fig. 5a) and α-lactose treatment did not offer significant protection to the mice (Fig. 5b).

HE staining revealed severe lung and spleen injury after infection with PbA (Fig. 5c and d). Histopathological scores were measured and both lung and spleen tissues showed significantly elevated scores, while there were no significant differences between the PbA and α-lactose treated group (Fig. 5e, f, left). Hemosiderosis was induced in mice lung tissue (Fig. 5c, marked in red), and this pathological sign seemed palliated after the treatment with α-lactose (Fig. 5c), the area of hemosiderin was reduced in the α-lactose-treated group compared to that of the PbA group, but the difference was not significant (Fig. 5e, right). Hemosiderosis was also observed in the spleen (Fig. 5d, marked in red); however, α-lactose treatment did not have a significant influence on spleen tissue (Fig. 5d, f, right).

**Fig. 6:**
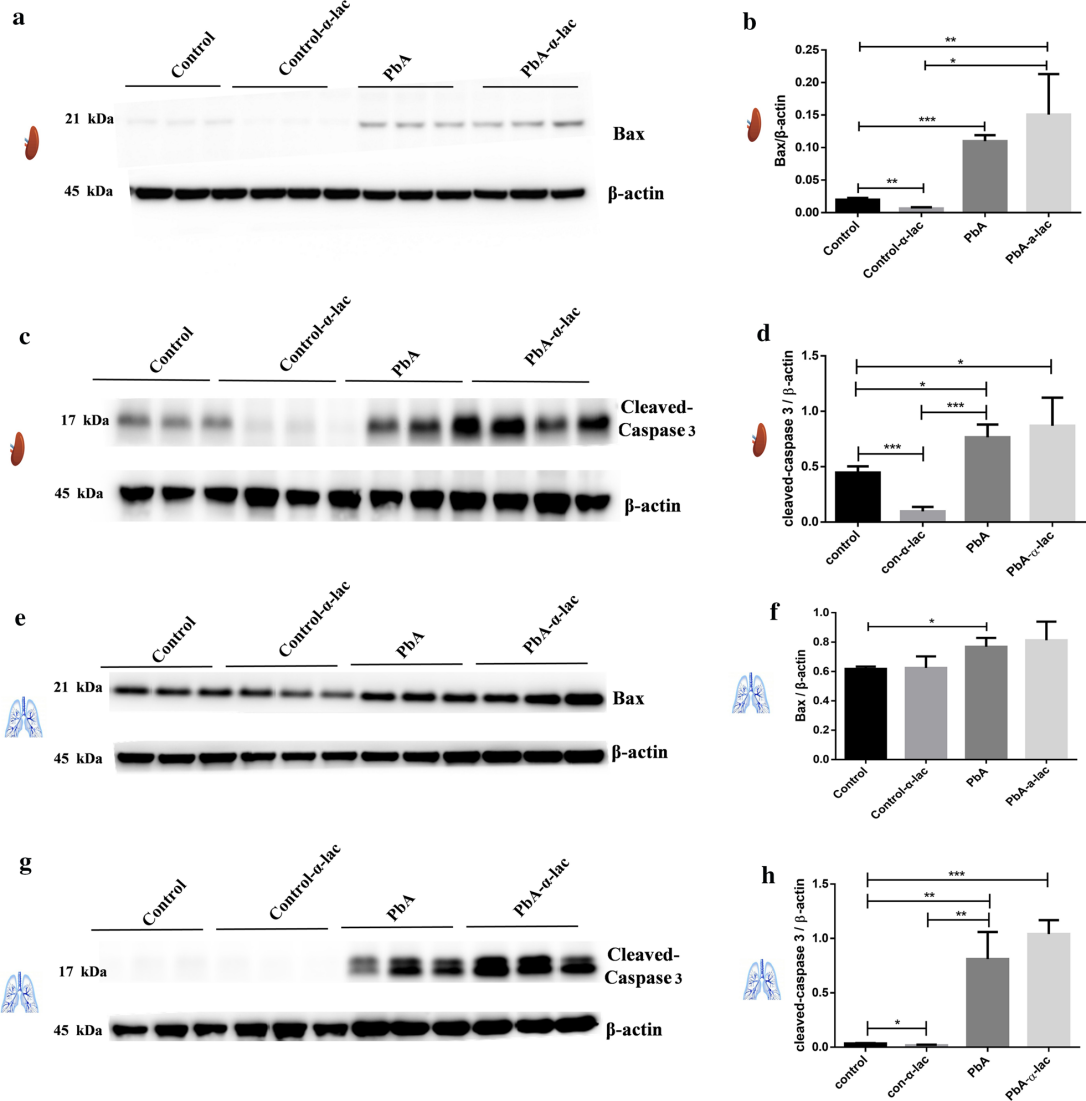
Western blotting results of lung and spleen tissues in *P. berghei* ANKA-infected mice with or without α-lactose treatment. (**a**–**h**) BAX and cleaved-Caspase 3 levels in mouse spleens (**a**–**d**) and lungs (**e**–**h**) were detected in Western blotting assays. β-actin was used as the control. Corresponding bands were scanned, and the optical density of the BAX or cleaved-caspase 3 bands were normalized by that of the β-actin band. The results are representative of three independent experiments with five to seven mice in each group per experiment, with data denoting means ± SDs. *P < 0.05, **P < 0.01, ***P < 0.001

Furthermore, the levels of BAX and cleaved-caspase 3 proteins in each group in both spleen and lung tissues were investigated by Western blotting assay (Fig. 6). Results showed that the levels of splenic BAX (Fig. 6a, b) and cleaved-caspase 3 (Fig. 6c, d) increased p.i., while there were no significant changes after α-lactose treatment (Fig. 6a–d). The level of BAX protein in lung tissue increased p.i. (Fig. 6e, f); however, α-lactose did not affect lung BAX levels (Fig. 6e, f). Interestingly, the levels of BAX and cleaved-caspase 3 proteins in spleen and the lung tissues all decreased after treatment with α-lactose in healthy mice (Fig. 6a–d, g, h).

### Gal-9-Tim-3 signaling blockade induced TIGIT expression in *P. berghei* infected mice

**Fig. 7:**
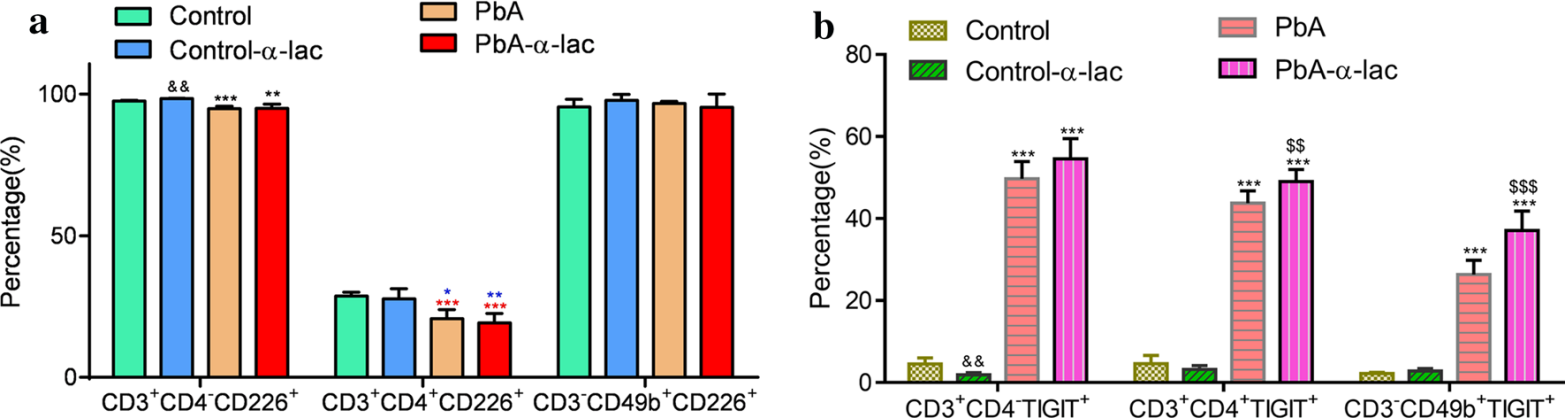
CD226 and TIGIT expression on lymphocytes of *P. berghei* ANKA-infected mice, with or without α-lactose treatment. The mice were sacrificed on 15 day p.i., and circulatory immune cells were obtained and analyzed using flow cytometry. **a** Histograms showing the proportion of CD226^+^ cells within the CD3^+^CD4^−^, CD3^+^CD4^+^, and CD3^−^CD49b^+^ cell populations among the four groups. **b** Histograms showing the proportion of TIGIT^+^ cells within the CD3^+^CD4^−^, CD3^+^CD4^+^, and CD3^−^CD49b^+^ cell populations among the four groups. The results are representative of three independent experiments with five to seven mice in each group per experiment, with data denoting means ± SDs. *^, $^ *P* <0.05, **^, $$, &&^ *P* <0.01, ***P < 0.001. *^(black)^ indicates comparisons with the control group and control-α-lac group. *^(red)^ indicates comparisons with the control group. *^(blue)^ indicates comparisons with the control-α-lac group. ^$^ indicates comparisons with the PbA group, and ^&^ indicates the comparisons with the control group

CD226, an Ig-like family glycoprotein expressed on a majority of immune cells, which can cause cellular activation [30], was analyzed. The proportion of CD3^+^CD4^−^CD226^+^ cells (Fig. 7a, left) and CD3^+^CD4^+^CD226^+^ (Fig. 7a, middle) cells decreased after PbA infection, and α-lactose treatment did not have a significant influence on the expression of CD226 (Fig 7a).

Therefore, the expression of TIGIT, which is a type of immune checkpoint receptor [31], was investigated using flow cytometry (Fig. 7b). The expression levels of TIGIT on CD3^+^CD4^−^ cells, CD3^+^CD4^+^ cells, and CD3^−^CD49b^+^ cells increased in PbA-infected mice compared with that in healthy mice (Fig. 7b). Interestingly, after treatment with α-lactose, the proportions of CD3^+^CD4^+^TIGIT^+^ cells (Fig. 7b, middle, *P *= 0.004) and CD3^−^CD49b^+^TIGIT^+^ cells (Fig. 7b, right, *P *< 0.0001) increased to higher levels in the α-lactose-treated group than those in untreated infected mice. This suggested that TIGIT expression increased in a compensatory manner after the blockade of the Tim-3-Gal-9 pathway, which might be one of the reasons for the eventual death of the mice.

## Discussion

T-cell immunoglobulin and mucin domain 3 (Tim-3) is a transmembrane protein expressed on many types of immune cells, including Th1 cells, NK cells, mast cells, macrophages, and DCs [32, 33]. Tim-3 signaling blockade in a murine model of experimental autoimmune encephalomyelitis (EAE) enhanced the pathological severity and clinical symptoms [34]. In lung tumor-bearing mice, anti-Tim-3 antibodies could prevent mice from developing resistance to anti-PD-1 treatment [19]. Furthermore, in patients with lung adenocarcinoma, higher levels of Tim-3, but not other immune checkpoint markers, were detected in T cells from patients who had developed resistance to anti-PD-1 therapy [19], indicating the importance of Tim-3. In *Plasmodium* infection, Tim-3 has been identified as an immune checkpoint molecule [13] and elevated percentages of T cell subtypes bearing Tim-3 were observed [20]. However, the dynamic expression of Tim-3 on key populations of lymphocytes during infection periods and its significance in disease resistance and pathogenesis has not been explored. Thus, we investigated the expression of Tim-3 on critical splenic and circulatory lymphocyte populations, as well as its regulatory role in lymphocytes exhaustion.

Our study revealed that the expression of Tim-3 on splenic CD8^+^ T cells (Fig. 1a, e), splenic CD4^+^ T cells (Fig. 1c, g), circulatory CD8^+^ T cells (Fig. 3a) and circulatory CD4^+^ T cells (Fig. 3b) cells in mice all increased, and the increase of Tim-3 on splenic CD8^+^ T cells, splenic CD4^+^ T cells, and circulatory CD4^+^ T cells was associated with a reduction in the proportion of these cells (Fig. 1i, m, k, o and Additional file 1: Figure S3a, c) during infection periods. The expression of Tim-3 on splenic CD49b^+^ cells increased to a peak level at 11 days p.i.; however, it declined towards the basal level in the subsequent days (Fig. 1b, f). This phenomenon was similar to the results of a previous study, which showed that FBXO38 mediated Lys48-linked poly-ubiquitination at the Lys233 site, leading to the degradation of PD-1 (also a well-known checkpoint) in the proteasome [35].

Studies have demonstrated the importance of cytokines for the efficient clearance of parasites during infection [36]. IFN-γ, which is produced by NK cells, among others, is essential for parasite clearance and could reduce the multiplication rate of *Plasmodium* [37]. TNF-α, which is predominantly secreted by activated macrophages [38], has been postulated to have antiparasitic effects [39]. Meanwhile, overproduction of IFN-γ or TNF-α could induce severe lung and liver injury [40]. In the present study, IFN-γ increased to its highest level at 4 days p.i., but then decreased (Additional file 1: Figure S6d), which might be resulted from the regulation of other anti-inflammatory factors, such as IL-4 (Additional file 1: Figure S6a), IL-6 (Additional file 1: Figure S6b), IL-22 (Additional file 1: Figure S6c), IL-10 (Additional file 1: Figure S6g), or IL-9 (Additional file 1: Figure S6h), and the decreased proportion of circulatory CD49b^+^ cells (Additional file 1: Figure S2b, d). The production of TNF-α increased p.i., which might result in the severe injury in lung and spleen tissues (Fig. 5c, d). IL-2, which is also a pro-inflammatory cytokine, was originally named T-cell growth factor (TCGF) for its capacity to enhance the proliferation and differentiation of T cells *in vitro* [41]. In the present study, IL-2 in mouse serum reached peaked at day 4 p.i. (Additional file 1: Figure S6f), and at the same time, the proportion of circulatory CD4^+^ T cells increased to their highest level (Additional file 1: Figure S3a, c). During *Plasmodium* infection, CD4^+^ T cells that produce IL-4 are essential for CD8^+^ T cells to control the parasite during the liver stage [42]. IL-6, which could facilitate B cell differentiation, is considered as an important component in the immune response against *Plasmodium* [43]. IL-22, of the IL-10 cytokine family, is originally produced by Th22 cells (a new line of CD4^+^ T cells) which could inhibit IL-4 production and plays an essential role in mucosal surface protection and tissue repair [44]. In *Il22*^−/−^ C57BL/6 mice infected with PbA, lower parasitemia was accompanied by a significantly earlier occurrence of cerebral malaria symptoms compared with that in wild-type mice [45]. Here, the levels of IL-4 (Additional file 1: Figure S6a), IL-6 (Additional file 1: Figure S6b), and IL-22 (Additional file 1: Figure S6) showed a similar trend during the infection period, indicating they might help to balance inflammatory reactions. IL-9 can enhance the survival of T cells, activate mast cells, and act in synergy with erythropoietin [44]. The function of IL-9 in *Plasmodium* infection has largely been unknown, and in this study, we found no significant changes in IL-9 expression p.i. (Additional file 1: Figure S6h). IL-10, which is produced by Th2 cells, inhibits the release of IL-2, IL-12, and IFN-γ, which would also decrease the antigen presentation capacity and MHC class II expression of DCs [44]. IL-10 has emerged as an important regulatory molecule with a protective role in experimental cerebral malaria (ECM) caused by *Plasmodium* in which it protects tissues by preventing excessive inflammation [46]. Meanwhile, in mice infected with *P. yoelii*, production of IL-10 and TGF-β were believed to correlate with high parasitemia and severe anemia [47]. Here, the IL-10 level was found to gradually increase after infection (Additional file 1: Figure S6g), which might result in decreases of several pro-inflammatory cytokines, leading to high parasitemia and severe anemia.

The spleen is very important for hematopoiesis and immuno-surveillance, and if a serious disorder occurs, spleen enlargement may be the first sign [48]. Studies have associated spleen enlargement with splenic infiltration by non-lymphocytes, for example, neutrophils [48]. We observed spleen enlargement p.i. (Additional file 1: Figure S4). Interestingly, the proportion of non-lymphocytes in the spleen increased sharply p.i. (Additional file 1: Figure S5a). Furthermore, the spleen index did not change in PbA infected mice after blocking the Tim-3-Gal-9 signaling pathway (Fig. 5a) suggesting that α-lactose cannot inhibit splenomegaly.

Tim-3 signal blockade using an anti-Tim-3 antibody elevated the activity of lymphocytes and improved the phagocytosis of macrophages, which resulted in accelerated clearance of the *Plasmodium* parasites in C57BL/6 mice, but did not prevent the final death of the mice [20]. In the present study, α-lactose, which is more convenient to obtain and cost-effective than anti-Tim-3 antibody, was used in BALB/c mice. However, α-lactose treatment showed neither significant protection (Fig. 5) nor prevented apoptosis of lung and spleen tissues in infected mice (Fig. 6). There were four distinct ligands reported to bind to Tim-3, which are galectin 9 (Gal-9), high-mobility group B1 (HMGB1), carcinoembryonic antigen cell adhesion molecule 1 (Ceacam-1) and phosphatidylserine (Ptdser) [49]. When PtdSer binds to Tim-3, it will influence the uptake of apoptotic cells and cross-presentation of antigen by dendritic cells [50]; the binding of HMGB1 to Tim-3 can suppress the activation of the innate immune response [51]; Ceacam-1 was identified as a novel cell surface ligand for Tim-3, the negative regulatory function of Tim-3 is defective in the absence of Ceacam-1 [52]. Our previous study showed that anti-Tim-3 antibodies which can literally inhibit the binding of all its ligands to Tim-3 could inhibit the growth of parasite and protect mice from cerebral malaria [20], while α-lactose only blocked the binding of gal-9 to Tim-3, which may be the reason why α-lactose did not protect mice as anti-Tim-3 antibodies did (Figs. 5 and 6) in this study. However, we found that the expression of TIGIT increased sharply p.i. and that blockade of Gal-9-Tim-3 signaling using α-lactose induced TIGIT expression in PbA infected BALB/c mice (Fig. 7b). TIGIT has been considered as an inhibitory checkpoint immunoreceptor. During *Plasmodium* infection, CD4^+^IFN-γ^+^IL-10^+^ T cells generally exhibit a short-lived effector activity and express high levels of TIGIT, an indication of cellular exhaustion [53]. Therefore, the compensatory expression of TIGIT in immune cells might be one of the causes leading to the ultimate death of the mice.

## Conclusions

An increased Tim-3 expression on splenic Tc, splenic Th and circulatory Th cells, accompanied by reductions in the proportions of these cells, was observed in *P. berghei* infected BALB/c mice. We concluded that Tim-3 on critical population of lymphocytes negatively regulates cell-mediated immunity against *Plasmodium* infection. Blocking Tim-3-Gal 9 signaling with α-lactose did not show significant protection to the mice; however, it induced the compensatory expression of TIGIT. The combination of blocking Tim-3 and TIGIT signaling may achieve a better protective effect, which need to be further investigated.

## Supplementary information

**Additional file 1: Figure S1.** Experimental protocol and scheme for drug treatment. **Figure S2.** Proportions of circulatory CD3^+^CD8^+^ cells and CD3^−^CD49b^+^ cells in mice infected with *Plasmodium berghei* ANKA. **Figure S3.** Proportion of CD3^+^CD4^+^ cells and CD3^-^CD19^+^ cells in mice infected with *P. berghei* ANKA. **Figure S4.** Spleen index of mice infected with *P. berghei* ANKA. **Figure S5.** Cell-type fractions across different tissues for mice infected with *P. berghei* ANKA. **Figure S6.** Cytokines production during infection of *P. berghei* ANKA.

## Data Availability

All relevant data supporting the conclusions of this article are included within the article and its additional files.

## Abbreviations

Tim-3: T-cell immunoglobulin and mucin domain 3
Gal-9: Galectin 9
α-lac: α-lactose
TIGIT: T cell immunoreceptor with Ig and ITIM domains
IL: interleukin
MHC: major histocompatibility complex
IFN-γ: interferon gamma
TFH: T follicular helper
NK: natural killer
PfEMP1: *Plasmodium falciparum* erythrocyte membrane protein 1
LAG-3: lymphocyte activation gene-3
CTLA-4: cytotoxic T-lymphocyte-associated protein-4
PD-1: programmed death-1
TNF-α: tumor necrosis factor alpha
PbA: *P. berghei* ANKA
i.p.: intraperitoneally
p.i.: post-infection
APC: allophycocyanin-conjugated
FITC: fluorescein isothiocyanate-conjugated
PerCP: peridinin chlorophyll protein complex-conjugated
BV510: Brilliant Violet 510™-conjugated
PE/Cy7: phycoerythrin/Cyanine7-conjugated
Ctrl: control
FACS: fluorescence activated cells sorting
FMO: fluorescence minus one
HE: hematoxylin-eosin
RPMI: Roswell Park Memorial Institute
PFA: paraformaldehyde
PBS: phosphate-buffered saline
HRP: horseradish peroxidase
EAE: experimental autoimmune encephalomyelitis
HMGB1: high-mobility group B1
Ceacam-1: carcinoembryonic antigen cell adhesion molecule 1
Ptdser: phosphatidylserine
