# Supplementary material for: Tim-3 signaling blockade with α-lactose induces compensatory TIGIT expression in Plasmodium berghei ANKA-infected mice
Source: Parasit Vectors. 2019 Nov 11;12:534. doi: 10.1186/s13071-019-3788-x (PMC6849286; doi:10.1186/s13071-019-3788-x)
Supplement: Supplementary file 1 — Additional file 1: Figure S1. Experimental protocol and scheme for drug treatment. Figure S2. Proportions of circulatory CD3+CD8+ cells and CD3−CD49b+ cells in mice infected with Plasmodium berghei ANKA. Figure S3. Proportion of CD3+CD4+ cells and CD3-CD19+ cells in mice infected with P. berghei ANKA. Figure S4. Spleen index of mice infected with P. berghei ANKA. Figure S5. Cell-type fractions across different tissues for mice infected with P. berghei ANKA. Figure S6. Cytokines production during infection of P. berghei ANKA. [file 13071_2019_3788_MOESM1_ESM.doc]

Additional file 1

**Tim-3 signaling blockade with α-lactose induces compensatory TIGIT expression in *Plasmodium berghei* ANKA-infected mice**

Yiwei Zhang^1,2†^, Ning Jiang^1,2†^, Ting Zhang^1^, Ran Chen^1,2^, Ying Feng^1,2^, Xiaoyu Sang^1,2^, Na Yang^1,2^ and Qijun Chen^1,2*^

^1^Key Laboratory of Livestock Infectious Diseases in Northeast China, Ministry of Education, College of Animal Science and Veterinary Medicine, Shenyang Agricultural University, Shenyang 110866, China

^2^The Research Unit for Pathogenic Mechanisms of Zoonotic Parasites, Chinese Academy of Medical Sciences, 120 Dongling Road, Shenyang 110866, China.

**^*^**Correspondence: qijunchen759@syau.edu.cn

†Equal contributors

E-mails:

YWZ: zyw@syau.edu.cn

NJ: jiangning1969@163.com

TZ: zhangting@stu.syau.edu.cn

RC: chenran@syau.edu.cn

YF: myfengying@syau.edu.cn

XYS: xysang2016@syau.edu.cn

NY: dayangna@syau.edu.cn

QJC: qijunchen759@syau.edu.cn


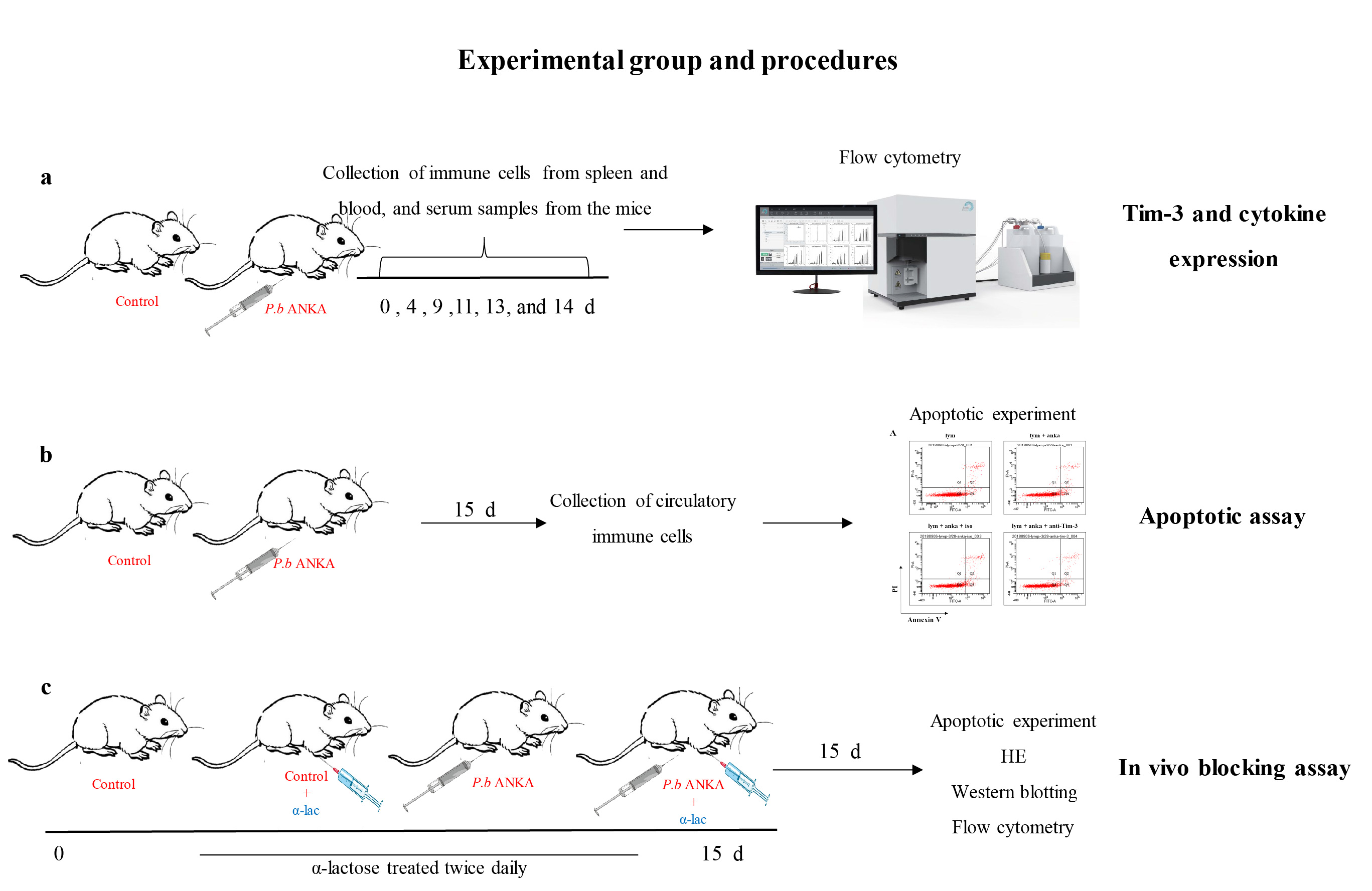


**Figure S1.** Experimental protocol and scheme for drug treatment.


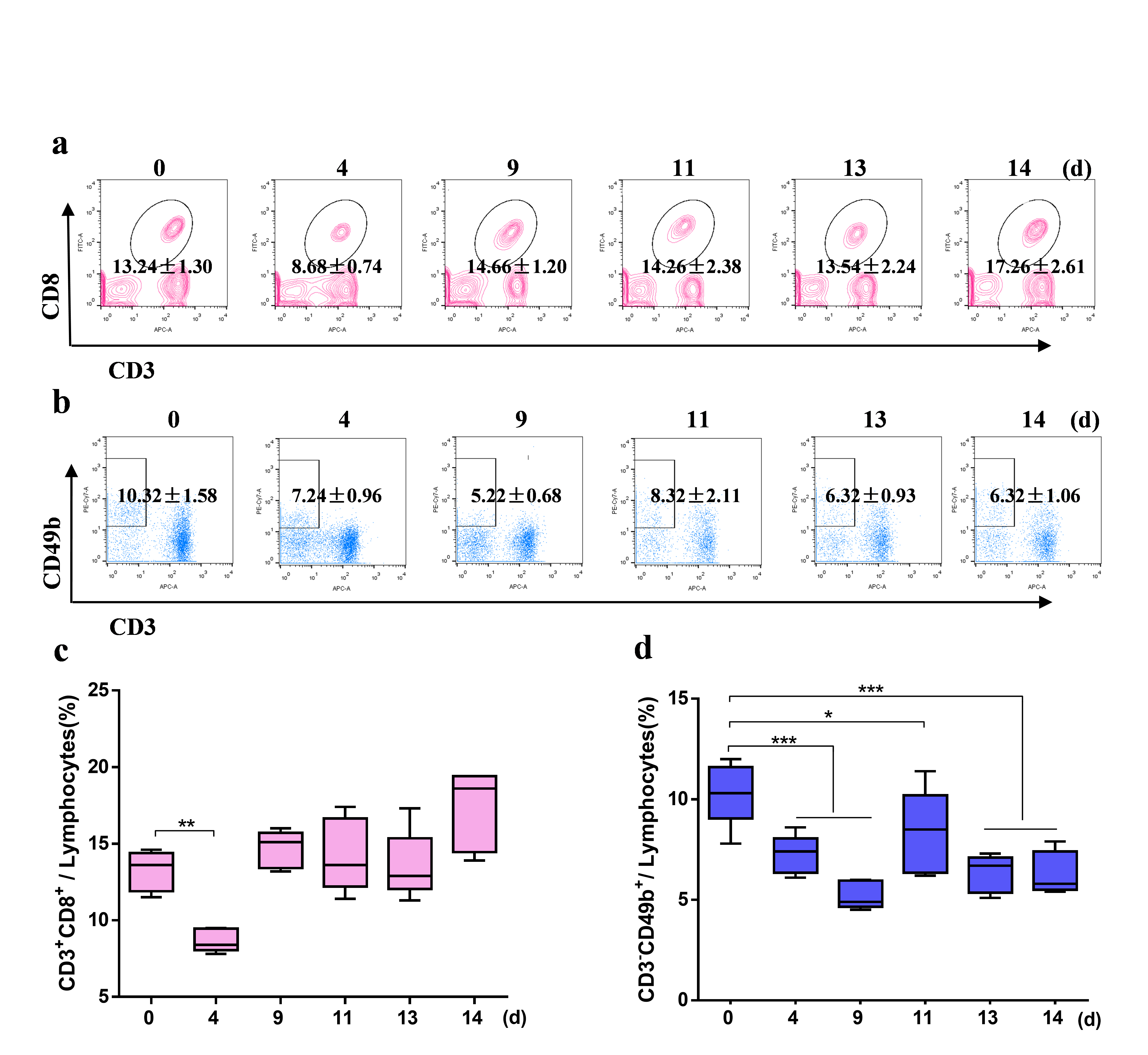


**Figure S2.** Proportions of circulatory CD3^+^CD8^+^ cells and CD3^−^CD49b^+^ cells in mice infected with *Plasmodium berghei* ANKA. Circulatory immune cells were isolated at 0, 4, 9, 11, 13 and 14 days p.i. **a** Representative FACS plots of CD3^+^CD8^+^ T cells in circulatory lymphocytes. **b** Representative FACS plots of CD3^−^CD49b^+^ cells in circulatory lymphocytes. **c** Box and whisker plots of the proportion of CD3^+^CD8^+^ T cells in circulatory lymphocytes. **d** Box and whisker plots of the proportion of CD3^−^CD49b^+^ cells in circulatory lymphocytes. The results are representative of three independent experiments with 5–7 mice in each group per experiment, with data denoting the mean ± SD. ^*^*P* < 0.05, ^**^*P* < 0.01, ^***^*P* < 0.001, ^*^ indicates comparisons with the day 0 group.


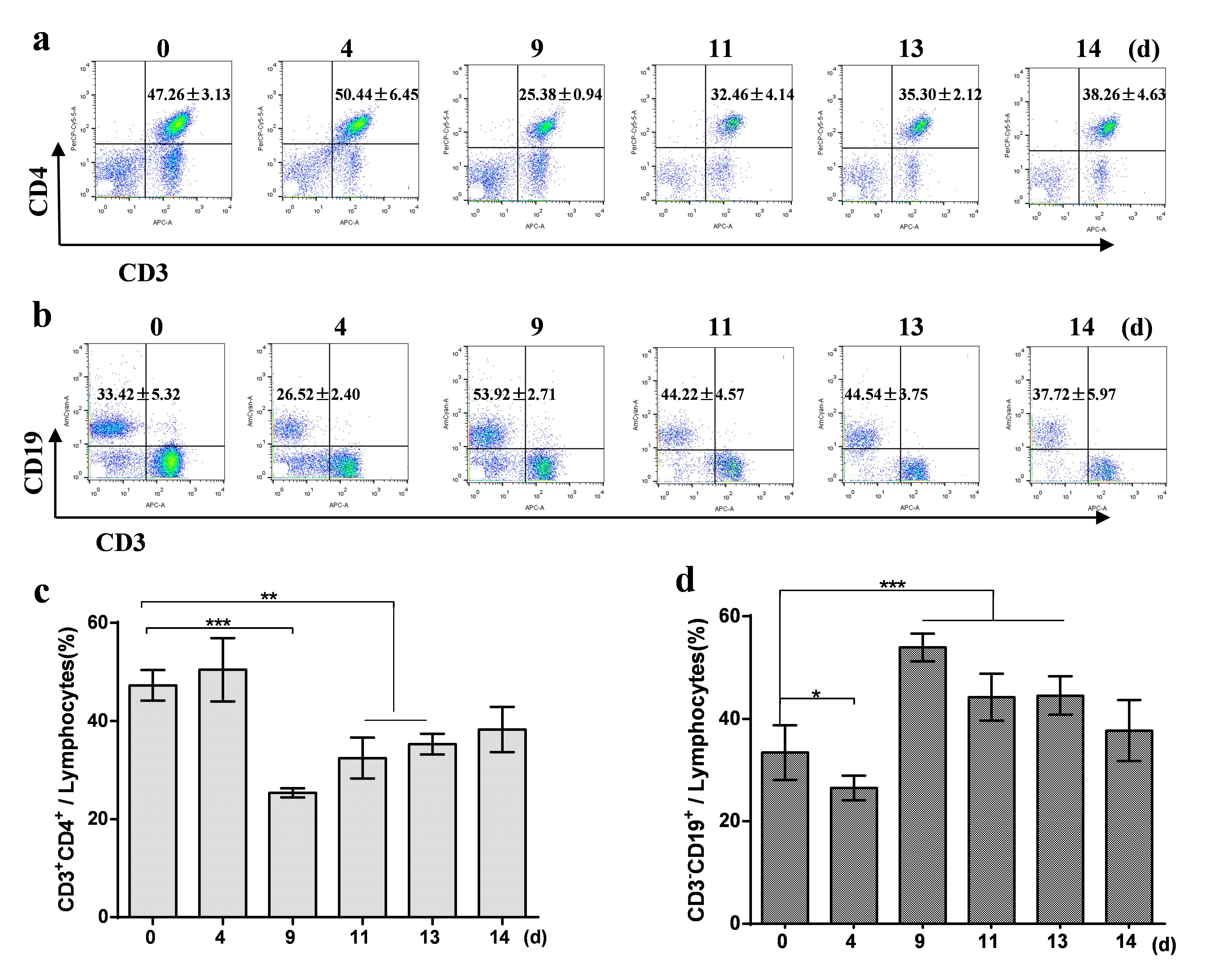


**Figure S3.** Proportion of CD3^+^CD4^+^ cells and CD3^-^CD19^+^ cells in mice infected with *P. berghei* ANKA. Circulatory immune cells were isolated at 0, 4, 9, 11, 13 and 14 days p.i. **a** Representative FACS plots of CD3^+^CD4^+^ T cells in circulatory lymphocytes. **b** Representative FACS plots of CD3^−^CD19^+^ cells in circulatory lymphocytes. **c** Histograms of the proportion of CD3^+^CD4^+^ T cells among circulatory lymphocytes. **d** Histograms of the proportion of CD3^−^CD19^+^ cells in circulatory lymphocytes. The results are representative of three independent experiments with 5–7 mice in each group per experiment, with data denoting the mean ± SD. ^*^*P* < 0.05, ^**^*P* < 0.01, ^***^*P* < 0.001, ^*^ indicates comparisons with the day 0 group.


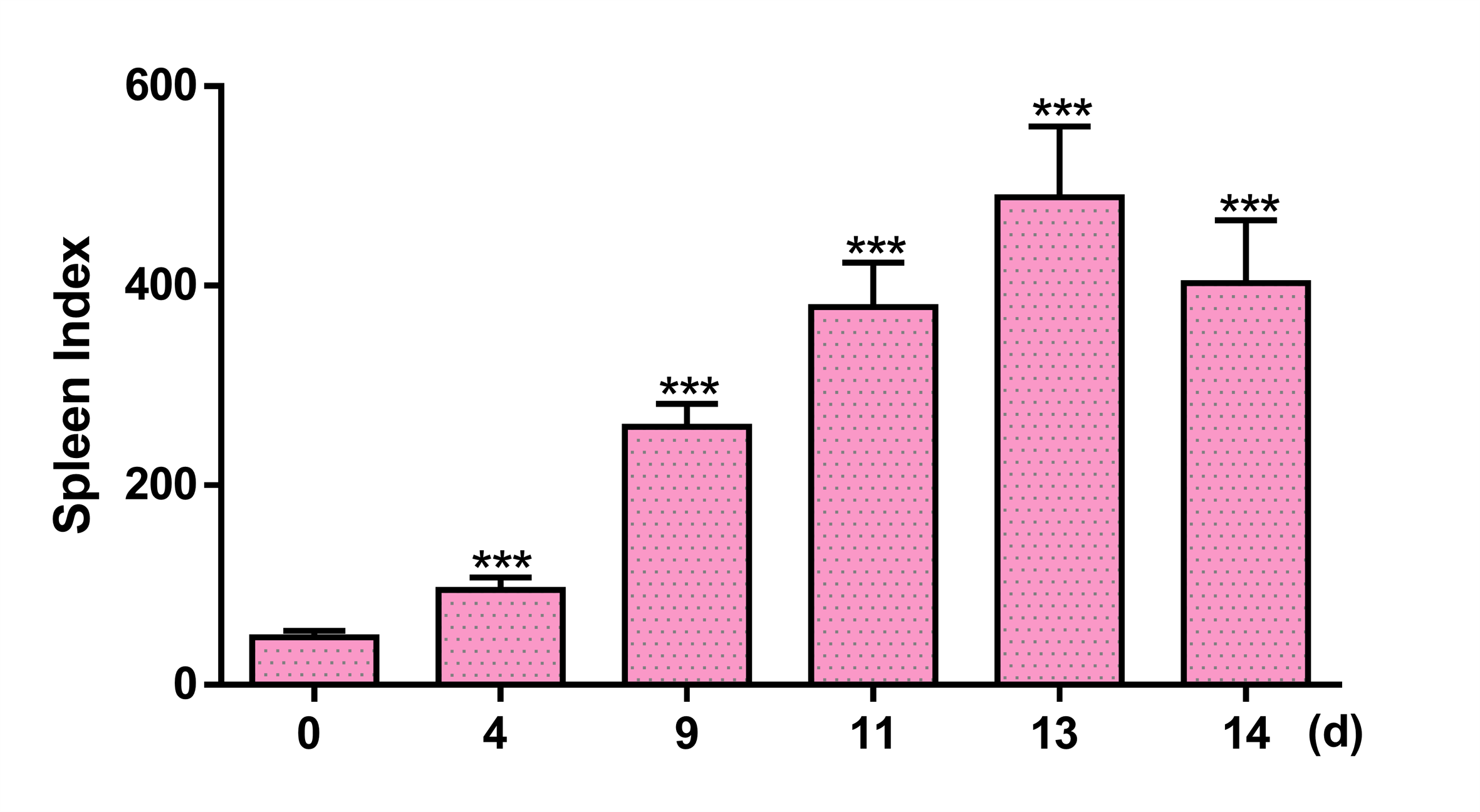


**Figure S4.** Spleen index of mice infected with *P. berghei* ANKA*.* Mice were sacrificed at 0, 4, 9, 11, 13 and 14 days p.i, and spleen indices were calculated. The results are representative of three independent experiments with 5–7 mice in each group per experiment, with data denoting the mean ± SD. ^*^*P* < 0.05, ^**^*P* < 0.01, ^***^*P* < 0.001, ^*^ indicates comparisons with the day 0 group.


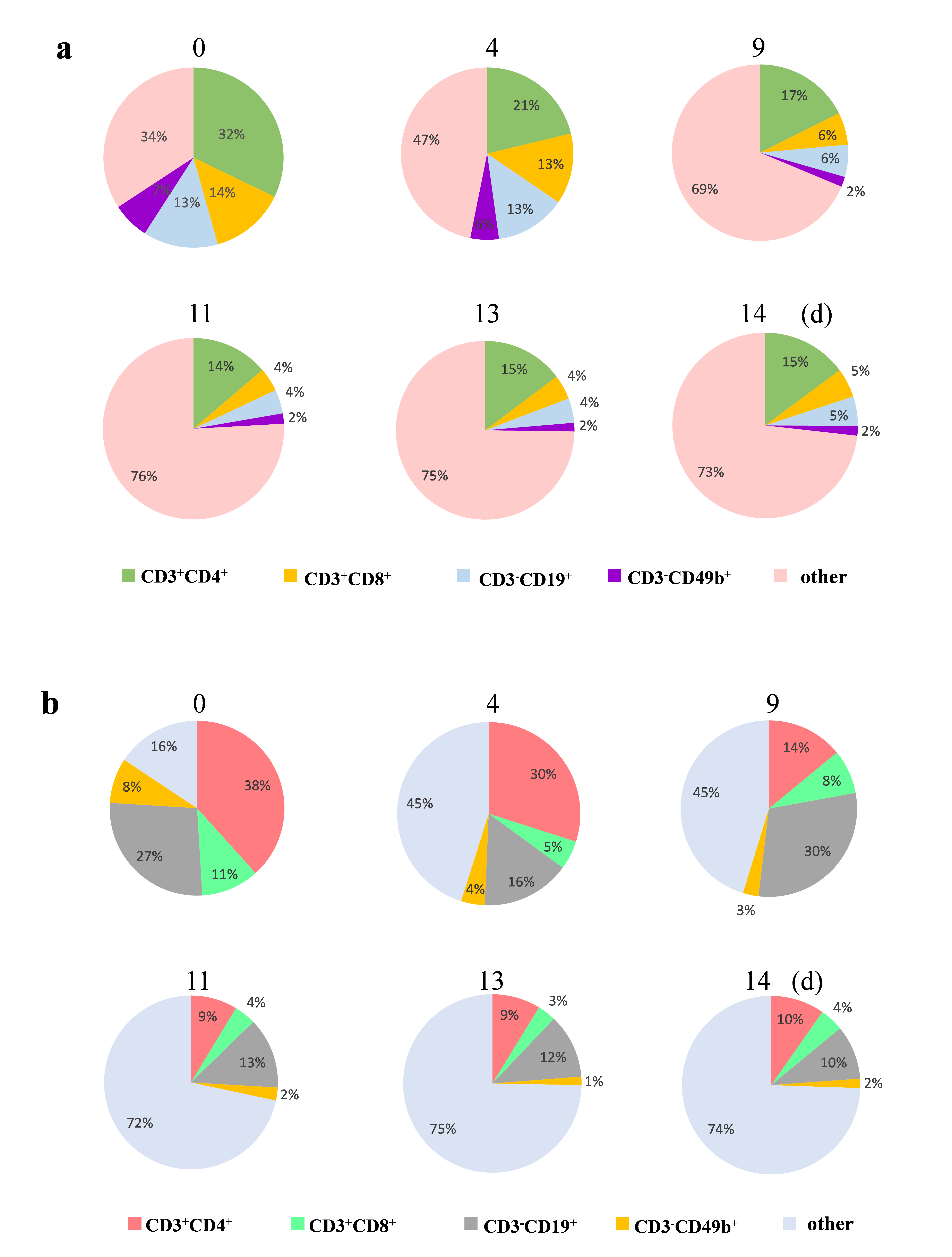


**Figure S5.** Cell-type fractions across different tissues for mice infected with *P. berghei* ANKA. **a** Pie charts of five cell types in splenic immune cells, colored by cell type. **b** Pie charts of five cell types in circulatory immune cells, colored by cell type. Values in the charts are the average of the data from 7 mice.


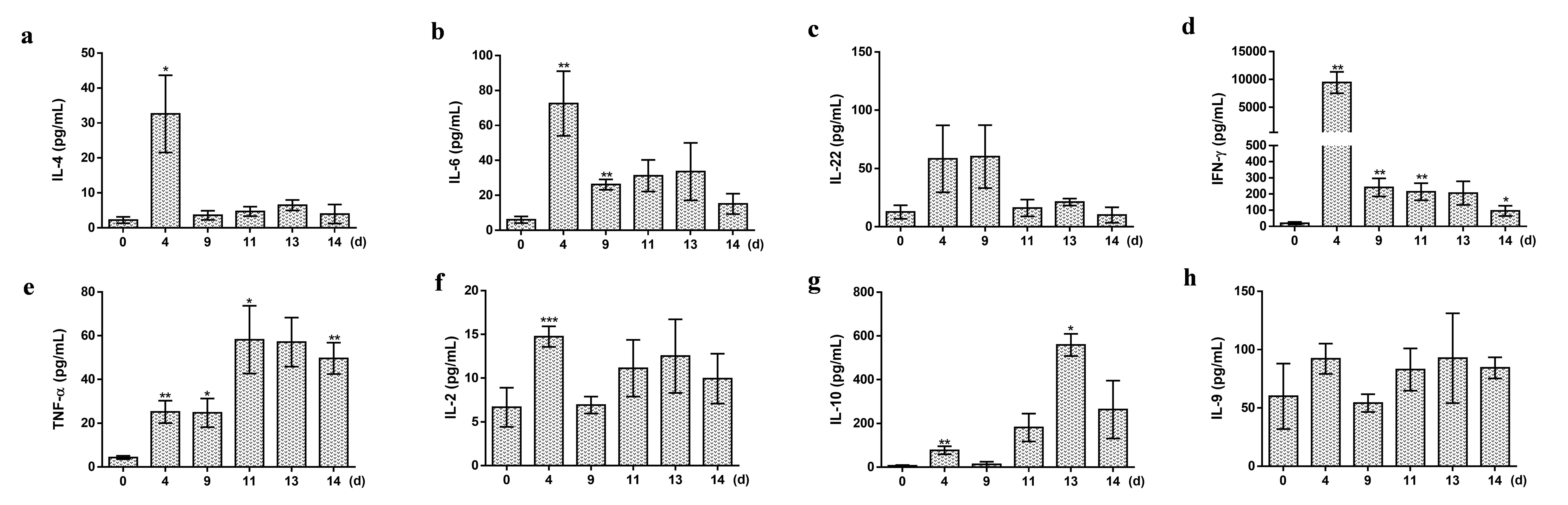


**Figure S6.** Cytokines production during infection of *P. berghei* ANKA. Cytokines were detected by flow cytometry using a LEGENDplex™ Mouse Th Cytokine Panel kit. **a**-**h** Changes in IL-4, IL-6, IL-22, IFN-γ, TNF-α, IL-2, IL-10 and IL-9, respectively, during infection. The results are representative of three independent experiments with 5–7 mice in each group per experiment, with data denoting the mean ± SDs. ^*^*P* < 0.05, ^**^*P* < 0.01, ^***^*P* < 0.001, ^*^indicates comparisons with the day 0 group.
